# Real-world implementation of the 2020 KDIGO guidelines for diabetes management in chronic kidney disease: a single-center retrospective study

**DOI:** 10.3389/fneph.2025.1664369

**Published:** 2025-09-15

**Authors:** Nomy Levin-Iaina, Hatem El’Nasasra, Anat Reiner-Benaim

**Affiliations:** ^1^ Department of Nephrology and Hypertension, Barzilai University Medical Center, Ashkelon, Israel; ^2^ School of Medicine, Faculty of Health Sciences, Ben Gurion University of the Negev, Beer Sheva, Israel; ^3^ Department of Epidemiology, Biostatistics and Community Health Sciences , School of Public Health, Faculty of Health Sciences, Ben Gurion University of the Negev, Beer Sheva, Israel

**Keywords:** type 2 diabetes mellitus, chronic kidney disease, KDIGO guidelines implementation, nephrology clinic, under treatment

## Abstract

**Background:**

Type 2 diabetes mellitus (T2DM) is an increasing global pandemic, frequently complicated by diabetic kidney disease, that may result in end stage kidney disease and increased cardiovascular morbidity and mortality. The 2020 KDIGO guidelines recommend SGLT2 inhibitors and GLP1RAs for cardio-renal protection in patients with T2DM and kidney disease. This study aimed to evaluate the implementation of the 2020 KDIGO guidelines among adult diabetic patients receiving nephrology care.

**Material and methods:**

This retrospective study included 587 patients with T2DM and chronic kidney disease treated in a single nephrology clinic between 1 May 2021 and 31 May 2022. Demographic, diabetes related, and CKD-related data was assessed. The utilization of the 2020 KDIGO recommended medications was analyzed during the study period, along with factors influencing treatment decisions.

**Results:**

The findings revealed a low initial utilization of recommended medications, with only 12.9% and 10.4% of patients treated with SGLT2i and GLP1RA, respectively. Only a modest, but significant, increase in SGLT2i usage was observed by the end of the study period. Factors associated with underutilization of SGLT2i and GLP1RA included older age and decreased kidney function. The study also highlights a significant gap between the recommendations given by nephrologists during the study period and the actual use of recommended medications in the last clinic visit.

**Conclusions:**

In conclusion, the study provides insights into the challenges of implementing KDIGO guidelines in real-world nephrology clinical setting. Further research is needed to explore the reasons behind low adherence to guidelines and strategies to improve compliance, ultimately enhancing patient outcomes in the management of kidney disease in T2DM.

## Introduction

Type 2 diabetes mellitus (T2DM) is a rapidly growing global epidemic, with a continuously increasing number of affected individuals worldwide (1). One of its most serious complications is diabetic kidney disease (DKD), a leading cause of chronic kidney disease (CKD) and end-stage kidney disease (ESKD) (2, 3). The global prevalence of DKD has risen significantly over recent decades, with an estimated annual incidence of 8% among patients with T2DM, and a 2 – 4% yearly decline in kidney function, culminating in an ESKD incidence of up to 0.8% annually (3).

Despite major advancements in glycemic control and cardiovascular risk management, the burden of DKD remains high and continues to grow (4, 5). DKD not only accelerates the progression to ESKD, but also significantly increases cardiovascular morbidity and mortality (6, 7). Optimal glycemic control plays a central role in preventing microvascular complications such as DKD (8–10), yet achieving and maintaining adequate glucose control remains challenging, particularly in patients with advanced kidney disease who face therapeutic limitations (11, 12).

For many years, standard DKD management has relied heavily on inhibition of the renin–angiotensin–aldosterone system (RAAS), particularly in patients with moderate-to-severe albuminuria (13, 14). In recent years, however, sodium-glucose co-transporter 2 inhibitors (SGLT2i) have emerged as disease-modifying agents with proven benefits in slowing DKD progression and reducing cardiovascular events when added to RAAS inhibitors (15–20). Similarly, glucagon-like peptide-1 receptor agonists (GLP1RAs) have demonstrated cardiovascular and potentially renal benefits in patients with T2DM and DKD (21–23).

In 2020, the Kidney Disease: Improving Global Outcomes (KDIGO) guidelines recommended SGLT2i for patients with T2DM and CKD (eGFR >30 ml/min/1.73 m²) as first-line agents for cardiorenal protection, and GLP1RAs as second-line therapy in patients not achieving glycemic targets with metformin and/or SGLT2i, or when those agents are contraindicated (24). These recommendations have since been endorsed by other leading professional societies (25–31).

Nevertheless, real-world evidence suggests underutilization of these agents remains widespread. In the United States, from 2017 to 2020, only 5.8% of adults with T2DM were treated with SGLT2i, and 4.4% with GLP1RAs, with little difference between patients with or without DKD (32). Similarly, in Canada, less than 8% of high-risk diabetic patients with DKD or cardiovascular disease received SGLT2i or GLP1RAs during the same period (33, 34).

To date, there is a lack of published data assessing the real-world implementation of the 2020 KDIGO guidelines, particularly in nephrology settings. Timely initiation of guideline-directed therapy in patients with DKD can significantly reduce the risk of kidney disease progression, ESKD, and cardiovascular events. Identifying gaps between evidence-based recommendations and actual prescribing practices is critical to improving patient outcomes.

The primary aim of this study was to evaluate the implementation of the 2020 KDIGO recommendations among adult patients with T2DM and CKD treated in a tertiary academic medical center nephrology clinic. Additionally, we sought to identify patient characteristics associated with the underuse of SGLT2i and GLP1RA therapies.

## Material and methods

### Study design and population

This was a retrospective, descriptive, single-center study conducted at the Nephrology Clinic of Barzilai University Medical Center. The study included adult patients (≥18 years) with a documented diagnosis of type 2 diabetes mellitus (T2DM) and chronic kidney disease (CKD) who attended the nephrology clinic between May 1, 2021, and May 30, 2022. The study period was selected to allow for at least one year to have elapsed since the publication of the 2020 KDIGO guidelines. Patients with type 1 diabetes mellitus or those receiving renal replacement therapy at baseline were excluded. CKD stage was defined based on eGFR or documented diagnosis in the medical records. For patients without available UACR during the study period, CKD diagnosis was based on prior laboratory results and documented medical records before study initiation. The study was approved by the Institutional Review Board and local Ethics Committee of Barzilai University Medical Center (approval number: 0057 - 22-BRZ 110809). Given the retrospective nature of the study, the requirement for informed consent was waived.

### Data collection

Patient data were extracted from electronic medical records and included demographic and clinical characteristics (age, sex, body weight, comorbidities), diabetes-related data (diabetes duration, HbA1c levels), kidney disease-related data (CKD duration, eGFR, serum creatinine, UACR, blood pressure, and underlying etiology of CKD) and additional laboratory data (lipid profile, hemoglobin levels).

Medication data were collected from the first and last nephrology clinic visits during the study period and included first-line antidiabetic agents (metformin, SGLT2i), second-line agents (GLP1RAs), other antidiabetic medications (DPP-4 inhibitors, insulin regimens) and cardiorenal protective agents (RAASi, lipid-lowering therapies, antiplatelet agents).

Recommendations made by the treating nephrologist regarding initiation or adjustment of diabetes and CKD-related therapies were also recorded.

### Statistical analysis

Baseline characteristics were summarized using means and standard deviations for continuous variables and counts with percentages for categorical variables. The proportion of patients receiving specific therapies was reported with 95% confidence intervals (CIs) and compared between the first and last clinic visits using McNemar’s test for paired categorical data.

Univariate comparisons between patients who did and did not receive guideline-recommended therapies (metformin, SGLT2i, or GLP1RA) were performed using the chi-squared test or Fisher’s exact test for categorical variables and independent t-tests or Mann–Whitney U tests for continuous variables, as appropriate.

To identify independent factors associated with the use of recommended diabetes therapies, a multivariate logistic regression analysis with stepwise variable selection was conducted. The model included baseline demographic, clinical, and laboratory variables. Results are presented as odds ratios (ORs) with 95% CIs. A two-tailed p-value <0.05 was considered statistically significant. All analyses were performed using R software, version 2023 (35).

## Results

### Patient characteristics

A total of 587 adult patients with T2DM and CKD who attended the nephrology clinic during the study period met the inclusion criteria. Baseline patient characteristics are described in **Table 1**. Patients had a mean age of 73.6 ± 10.3 years, and 62.7% were male. The mean number of nephrology visits during the study period was 2.05 per patient, with an average follow-up duration of 7.9 months between the first and last visits.

**Table 1:** Baseline patients characteristics according to recommended treatments with metformin, SGLTi and GLP1 RA.

		Total (n=587)	No Treatment (n=382)	Treatment (n=205)	P-Value
Baseline Characteristics					
Gender Age (mean) Weight (Kg)	Female Male Mean (SD) Mean (SD)	219 (37.3%) 368 (62.7%) 73.6 (10.3) 82.4 (17.2)	143 (37.4%) 239 (62.6%) 75.1 (10.3) 81.5 (16.7)	76 (37.1%) 129 (62.9%) 70.9 (9.8) 84.1 (18.1)	1.00 <0.0001 0.11
Background Diseases					
Hypertension Dyslipidemia Coronary Artery Disease Congestive Heart Failure		510 (86.9%) 350 (59.6%) 178 (30.3%) 95 (16.2%)	323 (84.6%) 191 (50.0%) 107 (28.0%) 70 (18.3%)	187 (91.2%) 159 (77.6%) 71 (34.6%) 25 (12.2%)	0.03 <0.0001 0.12 0.07
Diabetes Parameters					
Diabetes Vintage (years) HbA1C (%)	Mean (SD) Mean (SD) HbA1c<7% (n,%) HbA1C 7 - 8% (n,%) HbA1C 8 - 9% (n,%) HbA1C >9% (n,%)	17.2 (8.5) 7.4 (1.5) 82 (46.1%) 51 (28.7%) 21 (11.8%) 24 (13.5%)	18 (9.6) 7.4 (1.6) 46 (48.9%) 26 (27.7%) 10 (10.6%) 12 (12.8%)	16.4 (7.5) 7.4 (1.4) 36 (42.9%) 25 (29.8%) 11 (13.1%) 12 (14.3%)	0.25 0.86 0.87
Lipid Profile					
Total Cholesterol (mg/dl) Triglycerides (mg/dl)	Mean (SD) Mean (SD)	166.1 (66.1) 235.6 (255.4)	157.6 (69.2) 281.8 (354.8)	170.7 (65.2) 209.1 (176.4)	0.54 0.33
CKD Parameters					
CKD Vintage (years) CKD Stage CKD Cause	Mean (SD) G1 (eGFR>90 ml/min/1.73m^2^) G2 (eGFR 60 – 89 ml/min/1.73m^2^) G3A (eGFR 45 – 59 ml/min/1.73m^2^) G3B (eGFR 30 – 44 ml/min/1.73m^2^) G4 (eGFR 15 – 29 ml/min/1.73m^2^) G5 (eGFR<15 ml/min/1.73m^2^) DKD Hypertension/nephrosclerosis Glomerular diseases Cardiorenal syndrome Interstitial diseases Post-surgery MM/MGUS Obstructive uropathy NSAIDs/analgesic nephropathy Nephrectomy Post-AKI Drug-induced Unknown/multifactorial	7.7 (5.4) 40 (7.7%) 94 (18.0%) 121 (23.2%) 133 (25.5%) 91 (17.5%) 42 (8.1%) 279 (64.4%) 29 (6.7%) 11 (2.5%) 11 (2.5%) 9 (2.1%) 7 (1.6%) 2 (0.5%) 13 (3%) 5 (1.2%) 5 (1.2%) 2 (0.5%) 6 (1.4%) 54 (12.5%)	8.2 (5.7) 20 (5.9%) 53 (15.6%) 59 (17.4%) 92 (27.1%) 75 (22.1%) 41 (12.1%) 170 (60.3%) 20 (7.1%) 10 (3.5%) 10 (3.5%) 7 (2.5%) 4 (1.4%) 1 (0.4%) 9 (3.2%) 4 (1.4%) 2 (0.7%) 2 (0.7%) 2 (0.7%) 41 (14.5%)	7.0 (4.9) 20 (11%) 41 (22.7%) 62 (34.3%) 41 (22.7%) 16 (8.8%) 1 (0.6%) 109 (72.2%) 9 (6.0%) 1 (0.7%) 1 (0.7%) 2 (1.3%) 3 (2.0%) 1 (0.7%) 4 (2.6%) 1 (0.7%) 3 (2.0%) 0 (0.0%) 4 (2.6%) 13 (8.6%)	0.23 <0.0001 0.11
Kidney Function					
Serum Creatinine (mg/dl) eGFR (ml/min/1.73m^2^)	Mean (SD) Mean (SD)	1.8 (1.1) 46.6 (23.7)	2.1 (1.3) 42.3 (24.0)	1.4 (0.5) 54.6 (20.7)	<0.0001 <0.0001
Albuminuria					
Urinary ACR (mg/g)	Median (IQR) A1 (UACR <30mg/g) A2 (UACR 30 - 300mg/g) A3 (UACR >300mg/g) Nephrotic range (UACR>3000mg/g)	146 (557) 32 (14.7%) 99 (45.6%) 81 (37.3%) 5 (2.3%)	170 (643) 17 (13.0%) 58 (44.3%) 53 (40.5%) 3 (2.3%)	72 (347.3) 15 (17.4%) 41 (47.7%) 28 (32.6%) 2 (2.3%)	0.008 0.61

Treatment – metformin, SGLT2i, GLP1-RA. Treated and non-treated patients were compared univariately using Chi-squared test or Fisher Exact test for categorical variables, and t-test or Mann-Whitney test for continuous variables.

The majority of patients had hypertension (86.9%) and dyslipidemia (59.6%). Coronary artery disease and congestive heart failure were documented in 30.3% and 16.2% of patients, respectively. The mean duration of diabetes was 17.2 ± 8.5 years, and the mean HbA1c level was 7.4 ± 1.5%. HbA1c was <8% in 74.8% of patients and >9% in 13.5%. Blood pressure was <140/90 mmHg in 56% of patients; 11% had severely elevated blood pressure (>160/110 mmHg).

### Kidney disease profile

The mean duration of CKD was 7.7 ± 5.4 years. DKD was the most common etiology, accounting for 64.4% of known cases, followed by hypertensive nephrosclerosis. The mean eGFR was 46.6 ± 23.7 ml/min/1.73m². Most patients (66.2%) had CKD stages G3–G4, with 25.7% having earlier-stage CKD and 8.1% having stage G5 not on dialysis. UACR data were available for 35% of all patients. The median UACR was 146 mg/g (IQR 557). Among these, 85.3% had at least moderately increased albuminuria (UACR≥30 mg/g), including 2.3% in the nephrotic range (**Table 1**).

When restricting the analysis to patients eligible for SGLT2i and GLP1RA therapy (eGFR>29ml/min/1.73m²), UACR data were available in 53% of cases. In this subgroup, the median UACR was 120 mg/g (IQR 393). In contrast, patients with eGFR 20 - 29ml/min/1.73m^2^ demonstrated markedly higher values, with a median UACR of 320 mg/g (IQR 1077). Importantly, among those receiving guideline-recommended therapy, median UACR was significantly lower compared with untreated patients (72 mg/g vs. 170 mg/g, respectively, p=0.008) (**Table 1**).

### Utilization of guideline-recommended therapies

At the first nephrology clinic visit during the study period, the use of KDIGO-recommended antidiabetic therapies was notably low. Only 20.4% of patients were treated with metformin, and an even smaller proportion (12.9%) received SGLT2i. GLP1RAs were prescribed to just 10.4% of patients.

**Figure 1:**
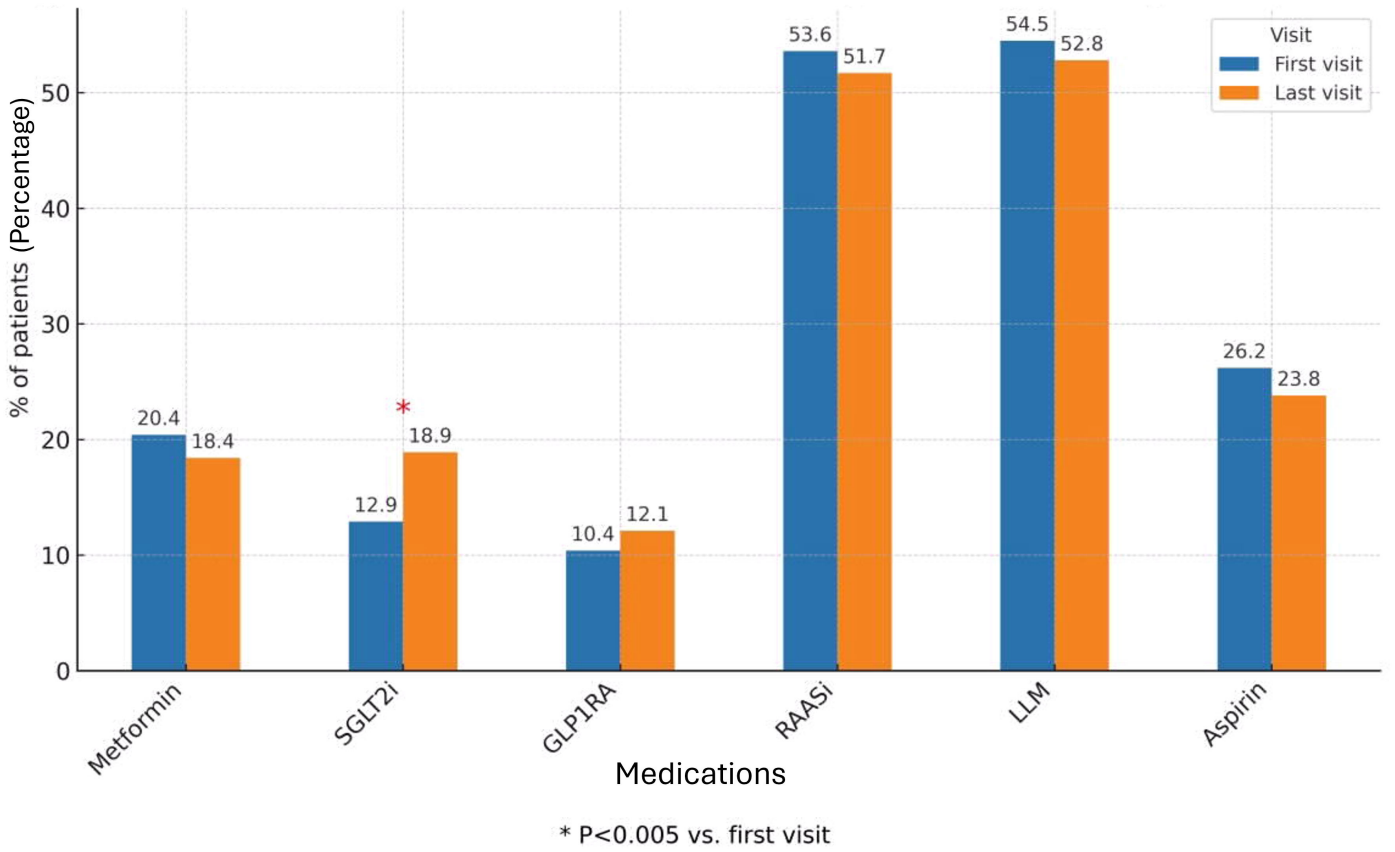
Diabetes and other CKD medications during first and last study visits (all cohort). The bar chart shows the proportion of patients treated with Metformin, SGLT2 inhibitors, GLP - 1 receptor agonists, RAAS inhibitors, lipid-lowering medications (LLM), and Aspirin at baseline (first visit) and at the last follow-up visit. Values are expressed as percentages of the total study cohort. The rates of treatment were compared between first and last visit during the study period, using McNemar’s test. The 0.05 level was used for significance. SGLT2i, Sodium-Glucose Co-transporter 2 inhibitors; GLP1RA, Glucagon-like peptide 1 receptor agonist; RAASi, Renin-Angiotensin-Aldosteron system inhibitor; LLM, Lipid lowering medication.

These low utilization rates persisted despite the fact that most patients were potentially eligible for such treatments: 74.4% had an eGFR above 30 ml/min/1.73 m², meeting the threshold for SGLT2i therapy, and 93% had eGFR above 15 ml/min/1.73 m², sufficient for GLP1RA initiation (**Figure 1**).

Additional glucose-lowering medications included DPP-4 inhibitors (10.6%), repaglinide (8.7%) and basal insulin (14.5%), while 3.2% of patients were treated with both basal and prandial insulin. Regarding other key cardiorenal therapies, 52.4% of patients were prescribed RAASi, 47.2% received lipid-lowering agents and 27.4% were treated with aspirin (**Figure 1**).

**Figure 2:**
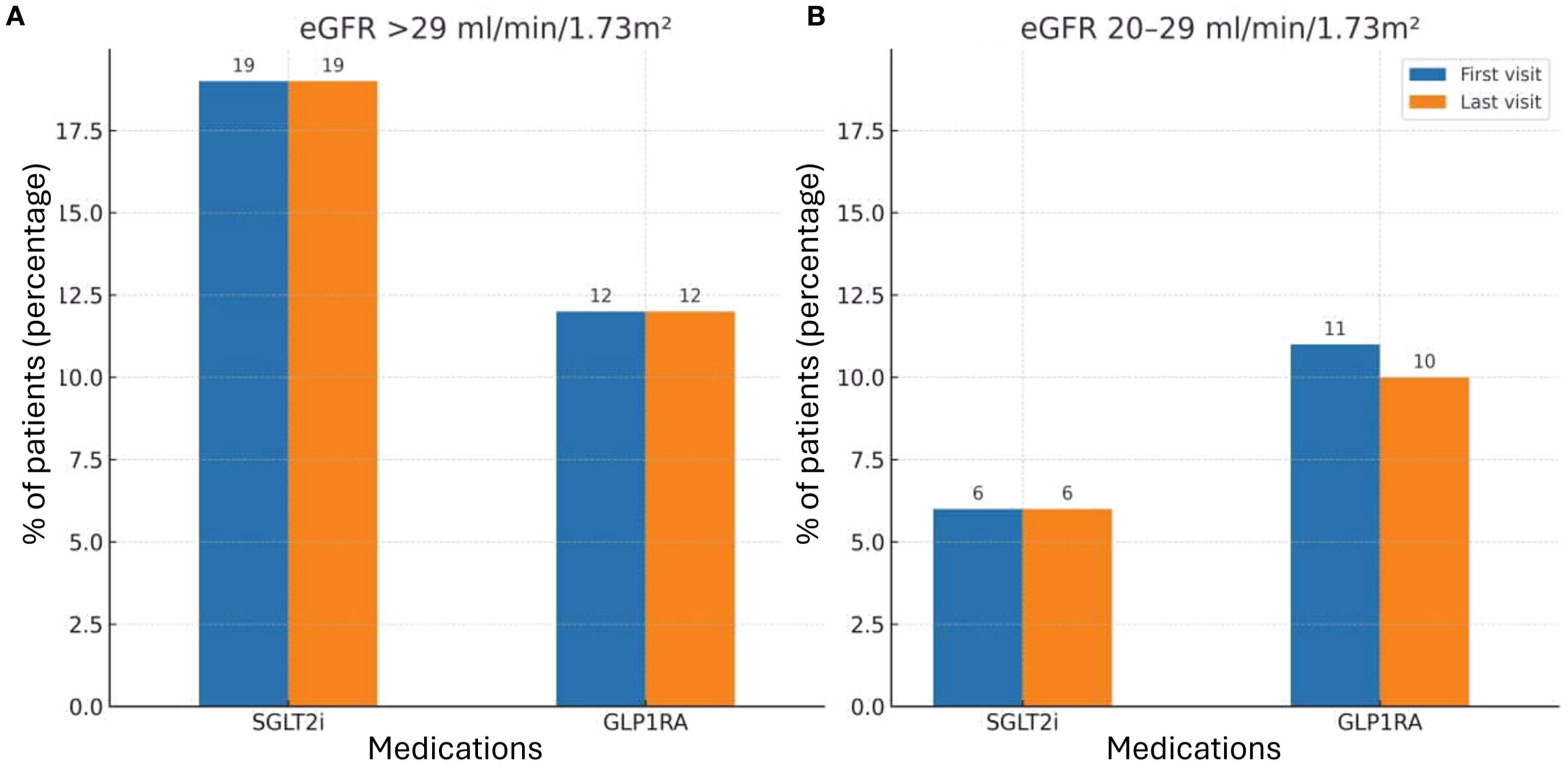
SGLT2i and GLP1RA treatment during first and last study visits by kidney function subgroups. **(A)** Patients with eGFR>29 ml/min/1.73m². Bars represent the percentage of patients receiving SGLT2i or GLP1RA at baseline (first visit) and at the last follow-up visit. **(B)** Patients with eGFR 20 – 29 ml/min/1.73m². Bars represent the percentage of patients receiving SGLT2i or GLP1RA baseline (first visit) and at the last follow-up visit. The rates of treatment were compared between first and last visit during the study period, using McNemar’s test. The 0.05 level was used for significance. SGLT2i, Sodium-Glucose Co-transporter 2 inhibitors; GLP1RA, Glucagon-like peptide 1 receptor agonist.

When stratifying patients by kidney function, utilization of SGLT2i and GLP1RA remained low in both subgroups (**Figure 2**). Among patients with eGFR>29 ml/min/1.73m² (**Figure 2A**), 19% were treated with SGLT2i and 12% with GLP1RA at the first clinic visit. In the subgroup with eGFR 20 – 29 ml/min/1.73m² (**Figure 2B**), only 6% received SGLT2i and 11% GLP1RA at baseline.

Throughout the study period, only 3.2% of patients received a nephrologist recommendation to initiate metformin. A recommendation to start SGLT2i was given to 15.2% of patients, while 6.5% were advised to initiate GLP1RA therapy. RAASi were recommended in 12.9% of cases. Importantly, of all the therapies evaluated, only SGLT2i demonstrated a statistically significant increase in utilization by the end of the study period, reaching 18.9% of patients (p = 0.005). In contrast, the use of metformin and GLP1RAs remained largely unchanged, underscoring a persistent gap between guideline-based recommendations and actual prescribing patterns (**Figure 1**). When analyzing the subgroups by eGFR categories, no difference was observed in treatment rates between the first and last study visits. Specifically, there was no increase in the use of SGLT2i across CKD subgroups, in contrast to the modest but significant rise noted in the overall cohort (**Figure 2**).

### Factors associated with the use of recommended diabetes medications

Univariate analysis comparing patients who received at least one of the KDIGO-recommended therapies (metformin, SGLT2i or GLP1RA) with those who did not, revealed several significant differences. Treated patients were generally younger, had better kidney function, as reflected by lower serum creatinine and higher eGFR and exhibited a higher prevalence of hypertension and dyslipidemia (**Table 1**).

Multivariate logistic regression analysis identified several independent predictors of therapy initiation. The presence of dyslipidemia was positively associated with the use of recommended medications (OR 3.11; 95% CI 1.96 - 4.94, p<0.0001). Conversely, older age (OR per year 0.95; 95% CI 0.92 - 0.97, p=0.0001), higher serum creatinine (OR 0.18; 95% CI 0.09 - 0.37, p<0.0001), and lower eGFR (OR 0.97 per ml/min/1.73 m²; 95% CI 0.95 - 0.99, p=0.0021) were independently associated with reduced likelihood of receiving recommended therapies (**Table 2**).

**Table 2:** Adjusted odds ratio for factors associated with recommended diabetes treatment in CKD patients.

Variable	Odds Ratio (95% CI)	P-Value
Age (years)	0.95 (0.92, 0.97)	0.0001
Dyslipidemia	3.11 (1.96, 4.94)	<0.0001
eGFR (ml/min/1.73m^2^)	0.97 (0.95, 0.99)	0.0021
Serum Creatinine (mg/dl)	0.18 (0.09, 0.37)	<0.0001

Adjusted odds ratios were obtained using multivariate logistic regression with stepwise model reduction procedure.

## Discussion

Diabetic kidney disease is a common and serious complication of type 2 diabetes mellitus, associated with an increased risk of ESKD, cardiovascular morbidity, and premature mortality (2). For decades, the management of DKD has focused on optimizing glycemic control, controlling blood pressure, and inhibiting the RAAS system. In recent years, the therapeutic landscape has evolved with the emergence of SGLT2i and GLP1RAs, which have demonstrated cardiorenal protective effects in patients with diabetes and CKD. Reflecting this evidence, the 2020 KDIGO guidelines position these agents as first- and second-line therapies for patients with T2DM and DKD (24).

The current study provides real-world evidence from a nephrology clinic setting, evaluating the implementation of the 2020 KDIGO guidelines approximately one year after their publication. Despite broad eligibility, we observed low rates of SGLT2i and GLP1RA use among patients with T2DM and CKD, both at baseline and at the end of follow-up. These findings are consistent with previous reports documenting a persistent gap between guideline-based recommendations and real-world prescribing practices (32, 33).

Most patients in our cohort had long-standing diabetes and significant comorbidities, including cardiovascular disease and hypertension, characteristics that place them at high risk for adverse outcomes and make them prime candidates for SGLT2i and GLP1RA therapy. Nevertheless, only 12.9% were treated with SGLT2i and 10.4% with GLP1RAs at the first clinic visit, despite eligible kidney function in the majority of patients. SGLT2i use increased modestly during the study period (to 18.9%, p=0.005), but the uptake of GLP-1 RAs remained unchanged.

Since, at the time of the study, guideline recommendations limited initiation of SGLT2i to patients with eGFR >29 ml/min/1.73m², we performed a subgroup analysis restricted to this cohort. Consistent with the main results, treatment rates remained very low, underscoring the substantial implementation gap. Following the subsequent KDIGO update extending eligibility to patients with eGFR ≥20 ml/min/1.73m², we also examined this subgroup. Similar to patients with higher eGFR, treatment rates in the 20 – 29 ml/min/1.73m² group were extremely low and showed no meaningful change throughout follow-up, further emphasizing the limited real-world adoption of guideline-directed therapy even among eligible patients.

Importantly, when considering only patients with eGFR>29 ml/min/1.73m², UACR data were available in 53% of cases, considerably higher than the 35% observed in the entire cohort. In this group, albuminuria levels were relatively lower compared to patients with eGFR 20 – 29 ml/min/1.73m², who exhibited much higher UACR values, consistent with more advanced kidney damage and greater cardiorenal risk. The observation that treated patients had significantly lower UACR compared with untreated patients suggests that appropriate initiation of guideline-directed therapy may be associated with reduced albuminuria burden, although causality cannot be established in this retrospective study.

Notably, even among patients for whom these therapies were explicitly recommended by the treating nephrologist, a substantial proportion did not initiate treatment by the last follow-up visit, highlighting potential barriers beyond the nephrology clinic, such as limited coordination with primary care or endocrinologists.

Our findings align with previous studies reporting underutilization of cardiorenal protective therapies in high-risk populations. For example, the multinational CAPTURE study (34) revealed low rates of SGLT2i and GLP1RA use, despite high cardiovascular disease prevalence, including in Israel, where these agents were prescribed to only 15% and 8.6% of patients, respectively. Similar trends were observed in national data from the United States and Canada (31–33).

In a study by Nargesi et al. (36), the use of SGLT2i and GLP1RA was evaluated among patients with diabetes and comorbid coronary artery disease, heart failure, or CKD, based on the treatment recommendations of the American College of Cardiology and the American Diabetes Association. Among the 1,104 patients included, 52.6% met the criteria for SGLT2i therapy, and 32.8% had an indication for treatment with a GLP1RA. Despite these indications, during the period between 2017 and 2018, only 4.5% received an SGLT2i and just 1.5% were treated with a GLP1RA.

In a later study from England (37) examined diabetes medication trends among ~150,000 primary care patients between 2017 and 2020, metformin remained the most commonly used drug (~62.5%), with minimal change over time. Although the use of SGLT2i increased from 6.7% to 13.8% in patients without cardiovascular disease, and from 4.3% to 20.1% in those with such disease, overall uptake remained modest. GLP1RA use rose only slightly, from 3% to 4.5%.

In a U.S. study by Limonte et al. (31), among 1,375 patients with T2DM (37% with CKD, average eGFR 88.4), only 5.8% received SGLT2i and 4.4% received GLP1RA. Among high-risk patients (CKD, heart failure, or CAD), usage was only 7.7% and 3.5%, respectively. Similarly, in a study by Hao et al. (38) of 7,168 diabetic patients in primary care (2018 – 2019), 77.7% were treated with metformin, 19.7% with SGLT2i, and 9.4% with GLP1RA. Among those with a cardiovascular indication (25% of the cohort), only 14.9% received SGLT2i and 4.6% received GLP1RA.

We identified in our study several factors independently associated with the use of recommended therapies. Younger age, higher eGFR, and lower serum creatinine were positively associated with treatment, while older age and impaired kidney function were linked to underuse. These findings echo previous literature indicating that physicians may hesitate to prescribe newer agents to older or frail patients, possibly due to concerns about adverse effects, polypharmacy, or perceived lack of benefit (31, 36).

Interestingly, we found a strong positive association between the presence of dyslipidemia and the use of metformin, SGLT2i or GLP1RAs. This may reflect a more proactive approach to cardiovascular risk management in patients already diagnosed with metabolic syndrome or established atherosclerotic disease.

Another important consideration is the persistence of insulin therapy without transitioning to guideline-recommended agents. In our cohort, nearly 18% of patients remained on insulin, with no adjustment toward SGLT2i or GLP1RA despite clear eligibility. Such therapeutic inertia is particularly concerning in insulin-dependent patients, as the continuation of insulin without modification is not easily justifiable. Insulin therapy carries well-documented risks, including weight gain, hypoglycemia, and local complications such as injection-induced lipohypertrophy, which increase both morbidity and healthcare costs (39).

Greater emphasis on proactive therapy modification, especially in collaboration with endocrinologists, diabetologists and primary care physicians, is warranted to reduce avoidable complications and to align real-world practice more closely with KDIGO guideline recommendations.

In addition to the underuse of diabetes-specific therapies, we also observed suboptimal use of long-established cardiovascular medications. Only 52% of patients received RAASi, 47% received statins and 27% were prescribed aspirin, despite decades of evidence supporting their role in reducing cardiovascular and renal events in high-risk populations. Suboptimal use of cardioprotective medications was also reported in other studies (34, 38). In the CAPTURE study (34), only 52% of patients received RAAS inhibitors, similar to the rate observed in our study, while 51% were treated with statins and just 39% with aspirin. These findings suggest that barriers to guideline implementation extend beyond newer agents and may reflect broader systemic issues.

Importantly, SGLT2i and GLP1RA therapies are reimbursed for most eligible patients, suggesting that cost or access limitations were unlikely to explain the low utilization observed. Instead, knowledge gaps, therapeutic inertia or lack of experience with newer agents, may play a role. Although our study did not explore physician-level attitudes or barriers, the discrepancy between nephrologist recommendations and actual implementation points to the need for better interdisciplinary coordination and follow-up.

To our knowledge, this is the first study to assess KDIGO 2020 guideline implementation in a nephrology clinic setting. Strengths include a relatively large sample size and detailed clinical data reflecting real-world care. However, the study has several limitations. As a retrospective analysis, we relied on patient-reported medication use, which may be subject to recall bias. We did not verify prescriptions or pharmacy dispensing records, nor did we assess medication adherence or reasons for treatment discontinuation. Although UACR was not available for the entire cohort, we performed subgroup analyses that partially mitigate this limitation, allowing more accurate assessment of albuminuria burden in the population eligible for treatments.

Taken together, our findings underscore a significant gap in the implementation of evidence-based therapies for patients with DKD. Bridging this gap requires targeted efforts to raise awareness among clinicians, improve interdisciplinary communication, and empower patients through education and shared decision-making. Ultimately, increasing the use of cardiorenal protective agents in eligible patients may reduce the burden of ESKD and cardiovascular complications in this vulnerable population.

## Data Availability

The raw data supporting the conclusions of this article will be made available by the authors, without undue reservation.
